# Social prescribing practices and learning across the North West Coast region: essential elements and key challenges to implementing effective and sustainable social prescribing services

**DOI:** 10.1186/s12913-023-09574-6

**Published:** 2023-05-31

**Authors:** Shaima M. Hassan, Adele Ring, Mark Goodall, Katharine Abba, Mark Gabbay, Nadja van Ginneken

**Affiliations:** 1 NIHR Applied Research Collaboration (ARC) North West Coast (NWC), Liverpool, UK; 2 The University of Liverpool, Liverpool, UK; 3 Brownlow Health, Liverpool, UK

**Keywords:** Social prescribing, Implementation, Health and wellbeing

## Abstract

**Introduction:**

Social prescribing has become an important feature of the UK primary care offer. However, there remains limited evidence on how best to implement and deliver social prescribing programmes to maximise effectiveness and long-term sustainability.

**Aim:**

To explore social prescribing practices and experience of implementing social prescribing programmes across National Institute for Health and Social Care Research (NIHR) Collaborative Leadership for Applied Health and Care Research (CLAHRC) North West Coast (NWC) and NIHR Applied Research Collaboration (ARC) NWC region to identify key learning points that can be applied to other settings.

**Method:**

We held a learning exchange workshop attended by practitioners and Public Advisors who had been involved in implementing and evaluating eight different social prescribing programmes with the support of NIHR CLAHRC NWC. We followed this with an online survey of social prescribing practice and priorities within the NIHR ARC NWC area. We used the findings from the workshop and survey to develop an initial model of the elements needed to successfully implement and sustain a working social prescribing programme.

**Findings:**

We identified three core essential elements for a successful social prescribing programme: a personalised approach; meaningful service-user and community involvement; and whole systems working. These core elements need to be supported with adequate resources in the form of continuity of funding and adequate community resources to refer people to, capacity building and appropriate evaluation.

**Conclusion:**

We were able to use a learning exchange workshop to both facilitate learning between practitioners and begin the process of identifying the ingredients needed for a successful social prescribing programme, which may be built on with further research.

**Supplementary Information:**

The online version contains supplementary material available at 10.1186/s12913-023-09574-6.

## Introduction

Unfair and avoidable differences in health and life expectancy between the most advantaged and most disadvantaged in UK society persist and in some instances are increasing [1]. Local government organisations in England are responsible for improving public health and reducing health inequalities with a key focus being the wider determinants of health [2], and healthcare staff also face increasing pressure to engage with the health inequalities agenda [3]. GPs spend around a fifth of their time on ‘social issues that are not principally about health’ [4] and it is recognised that social issues, such as debt or loneliness, can impact a person’s health and their ability to manage their health.

Non-medical approaches have long been considered an important adjuvant or alternative to medical treatment, and the importance of partnership between local government, the NHS and third sector organisations to develop non-medical interventions is recognised [5]. Recently these interventions have been collectively summarised as ‘social prescribing’ [6]. There are numerous definitions of social prescribing, most of which highlight the role of a link worker in supporting people to co-produce a ‘social prescription’ involving engagement in activities or connection with local community resources to improve their wellbeing [6]. There are many examples of social prescribing in action [6–9], with interventions ranging from basic sign-posting through to holistic support [10]. Although social prescribing is not a new construct [6], interest has vastly increased in recent years in line with the prevention agenda [11], increasing demand on primary health care services [12] and the urgent need to address long-standing health inequalities [13].

A critical discourse analysis published by Calderón-Larrañaga et al. in 2021 investigated how social prescribing is framed in scientific literature (89 references included) and explored its consequences for service delivery. Three types of discourse were identified: [14]

- *Discourse 1. Social prescribing as helping to overcome the social determinants of health.* Social prescribing functions as a referral pathway from health to community-based services. Assumes that the biomedical model of health services is unable to address social problems and aims to address the social needs of patients.
- *Discourse 2. “From dependence to independence”: Social prescribing as supporting patients' journey towards self-activation.* The rationale is to reduce the growing demand for healthcare resources by reducing healthcare utilisation. Social prescribing includes coaching and motivational interventions to enhance self-care and management of long-term illness. Assumes that general practice is overloaded and prioritises patients with the capacity to overcome problems by increasing ‘activation’, ‘self-efficacy’, ‘confidence’, ‘motivation’ etc. SP tends to be time-bound to prevent ‘dependency’ on the link worker. ‘Behaviour change’ is often the goal.
- *Discourse 3. Social prescribing as enhancing personalised care in general practice.* GP clinical appointments are seen as too rushed or impersonal to meet patients’ needs and expectations. Social prescribing is seen as a service to make up for these shortfalls by providing a more holistic and personal service. Targets people with complex and enduring health needs. Patients are moved back and forth across settings and sectors depending on their changing needs, requiring ongoing coordination between care providers. Knowing that support was available, as well as feeling listened to and cared for were sufficient and relevant endpoints.

Social prescribing is a key component of NHS England’s Comprehensive Model for Personalised Care [15], and currently all Primary Care Networks are contractually required to employ link workers to facilitate the signposting of patients to a variety of activities and support options [16].

Although there is limited evidence on the effectiveness of different social prescribing models, there is growing evidence to support the ‘pivotal’ role of the link worker [10, 17–20]. Recent reviews have also described factors facilitating engagement of patients in social prescribing (both in the co-production of the social prescription and in the activities ‘prescribed’). These include positive beliefs about the benefits of social prescribing, trust in the referrer and link worker, the context of the social prescribing, the way in which activities are presented by the link worker, perception of the link worker and activities as supportive, accessibility of activities and availability of support to attend activities [10, 19]. Barriers to engagement include fear of stigma, patient expectations, and the short-term nature of social prescribing programmes [19].

Understanding how social prescribing models ‘work’ is important for future commissioning of social prescribing services. Reviews of effectiveness have identified a plethora of different objectives, approaches, outcomes and outcome measures, adding to the complexity of determining what works [10, 17–19, 21, 22]. A recent editorial [23] proposed that, in order to generate useful evidence for the future, studies evaluating social prescribing should conceptualise social prescribing as a system, report contextual factors and their impact, and be realistic about what outcomes are relevant and useful.

In this paper we aim to add to the evidence base by collating, synthesising and interpreting data collected from people involved in social prescribing in the North West Coast area of England. Data included the models employed, the elements of those models viewed as core to their success, and the challenges involved in implementing a social prescribing programme and delivering a service that works. We drew on two complimentary sources, a ‘learning exchange workshop’ and an on-line survey, the content of which was informed by the workshop. The workshop and survey were both conducted through the National Institute for Health and Social Care Research (NIHR) Collaboration for Leadership in Applied Health Research and Care (CLAHRC) North West Coast (NWC) and its successor, the Applied Research Collaboration (ARC) North West Coast (NWC).

## Methods

### Context

The workshop and survey were conducted in early 2020 as part of the NIHR CLAHRC NWC legacy programme to produce research outputs from CLAHRC projects. CLAHRC NWC, the predecessor to the current ARC NWC, was a partnership between health and care service providers and universities conducting applied research and implementation to improve health and care for populations within the North West Coast area of England. The guiding principles of CLAHRC NWC were to reduce health inequalities and involve patients and members of the public in all of their work. These were supported by a pool of CLAHRC NWC/ARC NWC Public Advisors.

The CLAHRC NWC Partner Priority Programme (PPP) supported NHS and local authority partners to implement and evaluate new services or evaluate current services according to their own priorities. The main CLAHRC NWC research programme took forward research, evaluation and implementation projects prioritised by the Collaboration as a whole.

## Data collection

### Learning exchange workshop

We identified services that had been the subject of a CLAHRC NWC evaluation or implementation project and that also met the broad definitions of social prescribing (whether or not they were badged as ‘social prescribing’) through review of the CLAHRC NWC database. We therefore included any programme that supported people to access local community and other non-medical resources to improve their wellbeing or health. We identified eight projects, seven of which were involved in the CLAHRC NWC PPP.

We invited key contacts from each project, including the CLAHRC NWC Public Advisors involved to a half-day workshop, held at the University of Liverpool, to discuss and share learning from both the CLAHRC projects and experience in practice. We also invited three additional Public Advisors with an interest in social prescribing to help facilitate the workshop. The Public Advisors were included to ensure the inclusion of the public voice in the direction of the workshop discussions. The main aim of the workshop was for attendees to share learning; the collation of learning for research purposes was a secondary aim.

The workshop took place in February 2020 and was attended by 12 people, including four Public Advisors, who were together involved in eight CLAHRC NWC/ARC NWC projects. The workshop was facilitated by four academic researchers. A summary of the types of projects included in the workshop, and how they were evaluated, is shown in Table 1. All eight services included a link worker. Whilst some provided the social prescribing linking service alone (through a link worker or other connector), others offered wellbeing activities as well. Three operated a ‘hub’ model, where link workers and support activities were co-located. All three types of discourse [14] were identified (social determinants = 2; self-activation = 4; person-centredness = 2).

**Table 1:** Summary of CLAHRC/ARC NWC Social Prescribing projects included in the learning exchange workshop

**Project**	**Target Population**	**Primary Discourse** [14]	**Description**	**Evaluation**	**Service Model**
1 [24, 25]	Adults with low-level mental health needs	Person-centredness	A 14-week supported intervention to reduce loneliness and social isolation	*Approach:* Mixed methods *Outcomes:* Loneliness, isolation and wellbeing	Link worker plus (community connector and volunteer community champions)
2	Adults with experience of neurological trauma	Social determinants	Integrated multi-agency inpatient drop-in service set up to support the socioeconomic needs of patients	*Approach:* Mixed methods pre- and post- intervention evaluation *Outcomes:* Stress and anxiety	Service hub
3 [8, 26, 27]	Adults experiencing and recovering from mental distress	Self-activation	Community hub-based service offering support to people with mental distress on their recovery journey	*Approach:* Mixed methods *Outcomes:* Individual recovery and use of clinical services	Service hub and link worker
4 [28, 29]	Adults living with Motor Neurone Disease (MND)	Person-centeredness	Pilot project to identify community-based activities to support psychological wellbeing for people with MND	*Approach*: Qualitative *Outcomes:* Participants’ experience of the project and their thoughts about whether and how community-based activities could benefit people living with MND	Link worker plus (link worker and occupational therapist)
5	Women aged 18 and over in early stages of pregnancy	Self-activation	Pilot project to improve access to services and psychological health and wellbeing. Comprised peer support plus leaflet about local resources	*Approach:* Mixed methods study comparing the intervention vs information leaflet only *Outcomes:* Acceptability of the intervention, uptake of community and health resources, feelings of support, psychological health and wellbeing, feelings about their baby	Link worker plus (link worker and peer volunteers)
6	Young people aged 14 to 24 years with mental health difficulties	Self-activation	Integrated approach providing community-based social, psychological and health support for young people and their families	Approach: Mixed methods Outcomes: Access, engagement and mental health of young people	Resource/service hub and link worker (information, advice and guidance worker)
7 [30]	Adults with health and debt problems	Social determinants	GP-practice based initiative to reduce non-medical presentations by providing debt advice	*Approach:* Quantitative *Outcomes:* Various, including income maximisation and self-reported wellbeing	Case worker (debt advice service)
8	Adults	Self-activation	Proposed intervention mapping social networks of service users using an online tool and linking them with individualised local resources	N/A – it was not possible to implement the proposed intervention	Link worker

The workshop started with a presentation that: (i) introduced the topic of social prescribing, (ii) provided a brief summary of projects and proposed models and (iii) detailed the format for the day and questions for facilitated round table discussions. Attendees were asked to consider three social prescribing models that were represented in the CLAHRC NWC projects (active signposting, link worker, and resource/service hubs). They were then asked to share any facilitators and barriers they had experienced in implementing their intervention or service model, and what had been important in achieving their aims. Attendees were then asked to discuss three elements that the researchers had identified from the project reports as common across projects – easy self-referral, opportunity to express individual/personal needs, and opportunities to interact with similar others. Attendees were also asked what they thought were the other key elements in their projects. Finally, attendees discussed how they had evaluated their project, what impact the intervention or service had had and whether there was anything missing for them to be able to demonstrate effectiveness. The two discussion groups had a note taker to record key points to share with the wider group during a feedback session.

After the workshop, we gathered the notes from the discussion groups and feedback session. We correlated these with collated and summarized information on each project’s social prescribing activities and how they were evaluated, using CLAHRC project reports as source material. We also identified the primary discourse used around the project according to the classification system of Calderón-Larrañaga et al. (abbreviated to ‘social determinants’, ‘self-activation’ or ‘person-centredness’) [14].

We used the notes and our own reflections of the event to draft a summary of our initial impressions and interpretation of the group’s perceptions of key elements for implementation and sustainability of social prescribing services. We circulated this document to all attendees with a request for feedback. Responses were incorporated into the summary document and findings.

#### (i)* CLAHRC NWC/ARC NWC* Survey social prescribing survey

We conducted an online survey over an eight-week period between March 2020 and May 2020, using Google Forms. The questionnaire was informed by the discussion in the earlier learning exchange workshop. It included both fixed and free-text response questions on social prescribing programme components, referral pathways, community involvement, evaluation, and perceptions of the main challenges involved in delivering and evaluating social prescribing (see Additional file 1 / extra materials, for a copy of the questionnaire).

We sent an e-mail invitation with a link to the questionnaire to 88 people, including ARC NWC partners (representing various NHS and social care organisations) ARC NWC Public Advisors and other relevant contacts within the NWC region. Sixty-two of these people were known to us because they had registered to attend an ‘ARC NWC Social Prescribing Knowledge Exchange Event’ planned for April 2020. In the email we asked these contacts to forward the questionnaire to anybody they knew within the NWC region who were involved in social prescribing.

We analysed the fixed-choice responses using descriptive statistics within Google Forms, and analysed the free-text response qualitatively by grouping responses into themes.

### (3) Development of a visual representation

We used the main themes identified through the workshop and survey to develop a visual representation of the essential elements needed to implement a working and sustainable social prescribing programme. The model was developed through discussion across the team, and went through several iterations before a final version was agreed upon.

### Procedures for obtaining informed consent

This study is part of a NIHR CHAHRC NWC series of evaluation which included ethical approval for NIHR CLAHRC NWC Partners Priority Programme evaluation was obtained from the University of Liverpool Committee on Research Ethics (Ref:2236), Lancaster University for research on the CLAHRC-NWC evaluation (FHMREC17023); and University of Central Lancashire for research on the NIHR CLAHRC NWC Intern programme evaluation (STEMH608).

All methods were performed according to relevant guidelines and recommendations. We obtained informed consent from all participants prior to taking part in the overall evaluations.

Participants who have been previously involved in the overall evaluation where contacted for a follow up workshop. The letter inviting attendees to the workshop clarified the primary aim of the workshop was to share learning and that outcomes of the workshop would also be written-up for publication in an academic paper. This information was re-iterated at the start of the workshop, and we checked that all attendees were happy to participate. The workshop was not audio-recorded and no direct quotes from attendees were recorded verbatim or used in any publication.

The on-line questionnaire was headed by information explaining why the data were being collected and how they were used, followed by a question and tick box to indicate that they had read the information and were willing to take part in the survey. It was not possible to proceed with the questionnaire without filling in the consent box.

## Findings

### Learning exchange workshop

#### Core elements for successful implementation and sustainability of social prescribing

Workshop attendees identified three core elements as important for the implementation and sustainability of social prescribing programmes. We labelled these as (i) Adoption of *a personalised approach* (ii) embedding *public involvement* at all stages (iii) The development of *whole-systems working*. These concepts are further elaborated below.

*A personalised approach:* Attendees described two concepts that were central to a personalised approach, which we labelled as *mapping* and *engagement.* Mapping and development of detailed knowledge of the activities and support available was important to enabling service-users to be linked to local activities that met their needs. A detailed assessment of individual needs, preferences and potential barriers to engagement was important if service-users were to be linked to services and activities they could engage with. Practical strategies included co-developed checklists of activities and interests, and the use of theory-driven practice to facilitate personalised and motivating conversations between service-users and link workers. A personalised approach was important to all types of projects irrespective of the discourse around them.

*Public Involvement:* Several projects had active service-user forums that were involved in service development, and these were seen as essential to the development of the service. Involvement of local communities was viewed as central to the development of place-based services. Members of the public and service-users were also identified as having an important role in engaging others with local services and support. The need for better engagement with individual users of services was also emphasised, as ‘experts by experience’.

*Whole systems working:* Collaborative working was identified as central for delivering social prescribing and wellbeing activities, with an emphasis on longstanding partnerships that facilitated service development and delivery. Consistent with this approach was the role of the link worker in building relationships with local services and organisations. This was felt to be important in fostering two-way communication between link workers and other organisations. Hub-based models where some services were co-located or where there was space for delivery of services outside of the usual clinical setting was considered to support a more collaborative, holistic person-centred approach to service delivery.

### Internal Influences on implementation and sustainability

*Capacity:* Local capacity to deliver social prescribing and wellbeing activities was a concern. Increasing demands of the link worker role in the context of uncertainty around available training and support was noted. Mapping of local resources was a labour-intensive task in the context of growing numbers of referrals and increasing complexity of cases, and a need for protected link worker time for these tasks was identified. There was also concern about the capacity of local services and activities in the context of growing demand caused by referrals from link workers, and the need for training and support to be offered. There were unresolved questions as to how best to support the availability of the community resources on which social prescribing depends. Needs were identified for local leadership and co-ordination of social prescribing services to support the development of community resources and two-way communication pathways between social prescribers and those resources, and for the recruitment, training and support of more volunteers.

*Evaluation:* Most of the social prescribing programmes represented at the workshop were evaluated using both quantitative and qualitative approaches. Both process and outcome measures were used. Evaluation methods did not vary significantly between different types of discourses, though those with primarily a patient-centredness discourse focussed more on qualitative patient views, and those that were keen to broaden the primary care package to ‘bolt on’ social support had a greater focus on quantitative outcomes. Outcomes were measured using both validated (e.g. the Warwick-Edinburgh Mental Wellbeing Scale (WEMWBS)) and bespoke tools (e.g. goal-based measures developed with services users). A range of issues were identified in relation to evaluation, largely grouped within three main categories (Table 2). Other important challenges to evaluation included time (staff availability to undertake data collection, access to relevant data and time scale for evaluation) and costs (with cost-effectiveness evaluation noted to be resource-heavy and requiring substantial funding).

**Table 2:**
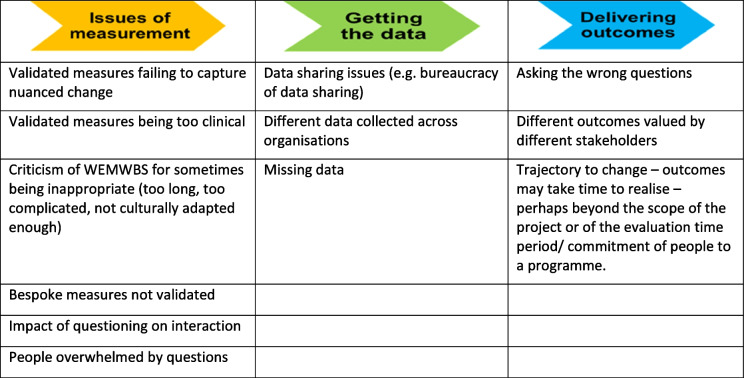
Perceived barriers to effective evaluation of SP programmes

### External influences on implementation and sustainability

#### Funding quantity and continuity

The need for long-term investment to support development and sustainability of local community resources was highlighted. Investment included funding to support training (e.g. preparation of funding bids—linked closely to capacity to deliver services and activities). Financial resources were considered to be limited and late in coming and it was noted that whilst link workers were paid, people running community groups and activities were often unpaid volunteers.

*Stakeholder support:* It was identified that the multifaceted nature of social prescribing meant there was need for leadership and co-ordination within the community. Stakeholder support, especially from commissioners, was considered important for implementation and long-term sustainability of social prescribing services.

### CLAHRC NWC/ARC NWC social prescribing survey

#### Respondents

The survey had 49 respondents, 34 (69.4%) of whom were actively involved in social prescribing, 15 (30.6%) of whom were not directly involved but were aware of social prescribing programmes. Respondents who were active in social prescribing identified themselves as link workers (*n* = 11), managers (*n* = 17) and commissioners (*n* = 6). The respondents provided information about 50 social prescribing programmes. Each programme was described by only one respondent.

#### Geographic areas and target populations

**Fig. 1:**
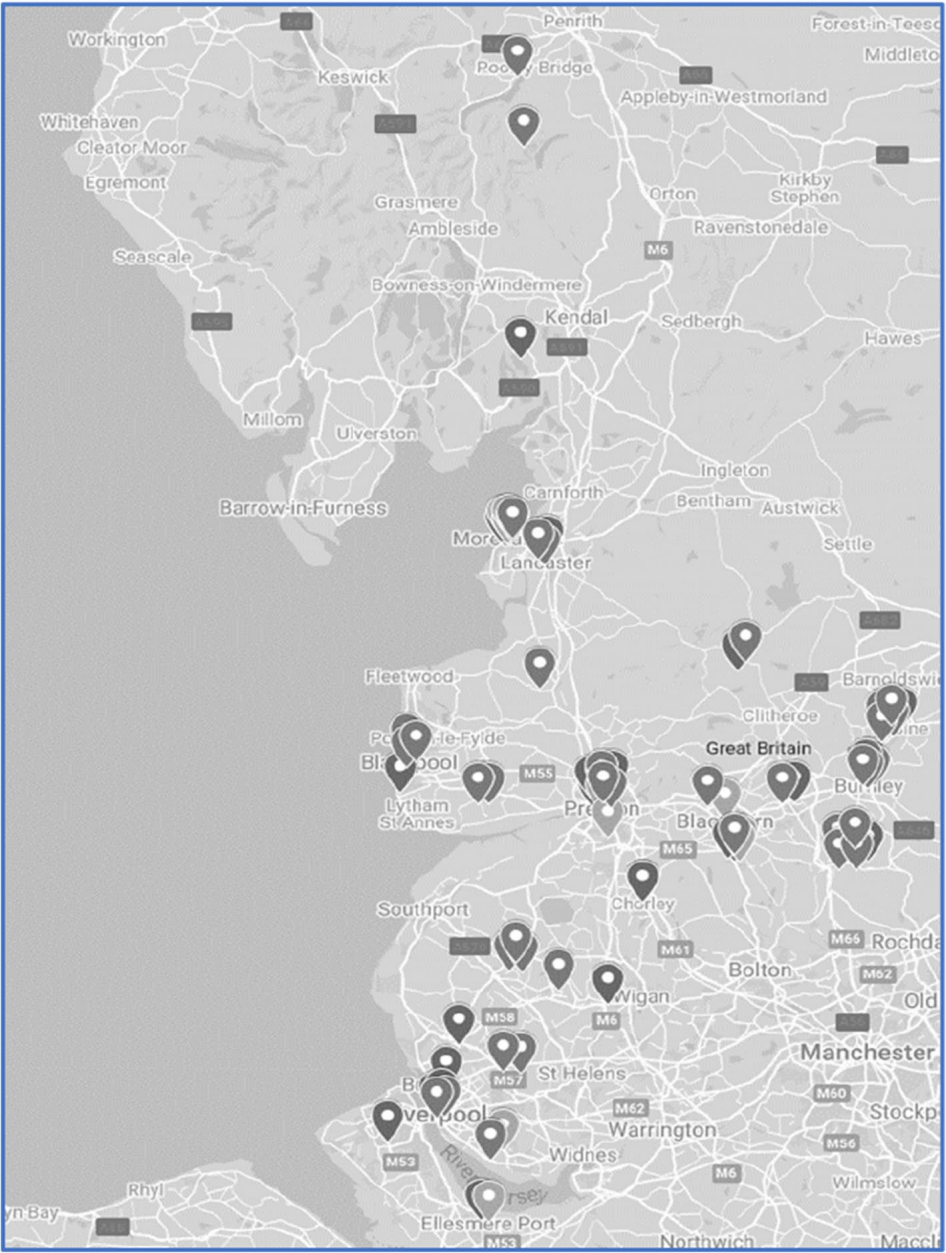
Geographical distribution of the NW Coast social prescribing projects surveyed

Of the 50 programmes identified across NW Coast region, 47 (98%) covered urban areas, 28(56%) covered semi-rural areas and 33 (66%) covered rural areas (Fig. 1). Most programmes were available to adults of all ages (*n* = 40), around a third (*n* = 16) included children and 3 were specific to adults above retirement age.

#### Programme aims

The stated aims of the included programmes were diverse; encompassing the very specific, e.g. ‘to reduce alcohol related self-harm’ and the very broad, e.g. ‘to reduce health inequalities’.

#### Models of social prescribing

The majority (38, 76%) of programmes had a reported role of ‘linking people to activities’ and half (25, 50%) were ‘delivering activities’. A role akin to the link worker model was commonly identified, including ‘link workers’ (*n* = 22), ‘community navigators’ (*n* = 7) and ‘community connectors’ (*n* = 12), suggesting that in fact at least 41 (82%) of the programmes in the survey sample were engaged in assessment, preparation and signposting activities. Other individuals and groups delivering social prescribing services included: case workers, GPs, nurses, dietician, community groups, volunteers, teachers, health trainers and care co-ordinators.

Seven main routes into services were reported, based on the options provided in the questionnaire, with access via general practitioners being the most common route. Self-referral was also common with 60% of respondents reporting a self-referral option (Table 3).

**Table 3:** Pathways into social prescribing and wellbeing services

Access routes	Social Prescribing projects surveyed *n* = 50
Primary Care – GPs	38 (76%)
Self-referral	30 (60%)
Community Health	27 (54%)
Social Care	25 (50%)
Secondary Care—Mental Health Services	24 (48%)
Drop in	22 (44%)
Secondary care—Physical Health Services	21 (42%)

Other access routes described: Community volunteers; Third sector; Community Police, Fire; Families and friends; Maternity; Social Housing Association

#### Public involvement

Respondents reported public involvement in 33 (66%) of the programmes, and indicated a range of public involvement roles from the options provided in the questionnaire, including:

- Raising awareness of the service (*n* = 23)
- Evaluation (*n* = 22)
- Co-creation of the service (*n* = 19)
- Monitoring (*n* = 16)
- Delivery of services (*n* = 15)
- Designing modifications to the service (*n* = 11)

#### How services were evaluated

Of 50 projects, 29 (69%) reported undertaking service evaluation. Across projects both quantitative and qualitative methods were being used. Almost a quarter of programmes reported using their own data reporting measures.

A range of challenges to service evaluation were reported. Based on the options provided in the questionnaire ‘determining what outcomes to measure or data to collect’ was the challenge reported by most respondents (69%), followed by ‘Capturing and accessing’ the data and ‘finding the time for evaluation’ (Table 4).

**Table 4:** Evaluation challenges

Challenge	Social Prescribing projects surveyed *n* = 29
Deciding what outcomes to measure or data to collect	20 (69%)
Capturing or accessing the data you need	15 (52%)
Finding the time for evaluation	14 (48%)
Analysing the data	6 (20%)
Satisfying the funders’ reporting requirements	6 (20%)
Interpreting the findings	3 (10%)
Making changes based on the findings	3 (10%)

#### Long-term sustainability of services

The majority, 35 (70%), of programmes had been active for three years or less at the time of the survey, and the majority reported having continued funding for less than two years.

#### Important elements for success

When asked what was important for the success of social prescribing initiatives, from a list of options, the majority (31, 62%) reported the need for adequate and sustainable funding (Table 5).

**Table 5:** Perceived priorities of SP project success

Key elements	Social Prescribing projects surveyed *n* = 50
Securing adequate and sustainable funding	31 (62%)
Getting the programme set up and running	21 (42%)
Engaging or motivating people to engage in services or activities	21 (42%)
Developing skills or capacity – community support or activities	10 (20%)
Developing skills or capacity – linking people into activities	9 (18%)

Other factors identified included building and maintaining relationships, targeted or generic social prescribing, social or medical model, communication, data sharing, operationalising activities, service buy-in to voluntary service opportunities and workload of people with complex needs

### Synthesis of findings into a visual representation

**Fig. 2:**
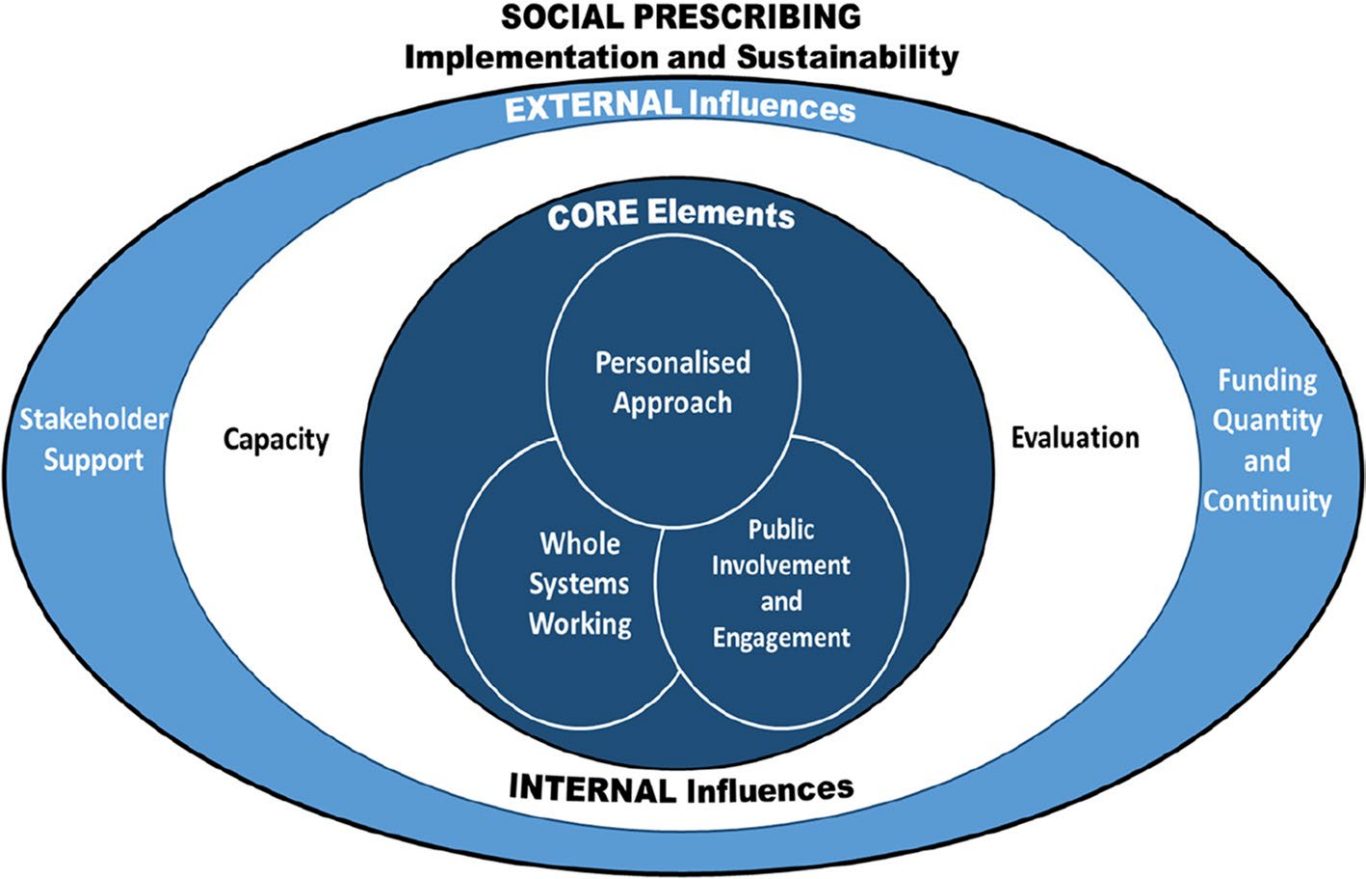
Social prescribing elements and influential factors on implementation and sustainability

A visual representation of the core elements, internal influences and external influence required for successful implementation, delivery and sustainability of social prescribing is presented below (Fig. 2). This representation highlights three core characteristics identified as essential to a successful social prescribing programme: adopting a personalized approach; working holistically across the whole system; and involving service-users and/or local communities in a meaningful way. Beyond these core characteristics, an ‘internal influences’ ring highlights the importance of adequate capacity within the system (both the social prescribing linking service and the community resources it depends on) to meet the needs of the population, and appropriate service evaluation, to the enactment of successful social prescribing. Further external influences on successful social prescribing include secure and adequate funding and the support of wider stakeholders, including commissioners.

## Discussion

### How our findings compare with current recommendations and other research findings

#### Types of services provided

We found considerable diversity in the focus and operation of social prescribing programmes within the North West Coast area. Although GP practices were the most frequently-reported referral route into social prescribing, other routes were available including from secondary care and social care, via self-referral, and as a ‘drop in’. This wide range of referral routes (assuming adequate coverage and resources) is in line with NHS recommendations to maximise accessibility of social prescribing by providing ‘systems that enable a broad range of agencies and organisations to refer in, as well as easy self-referral processes’ [15]. The heterogeneity of the social prescribing programmes in terms of their clients groups and objectives is consistent with other UK-based research [31]. This diversity has been explained previously as resulting from the ‘demand-driven formalisation of referrals to existing community services and organisations which is necessarily locally different’ [10].

#### The elements and conditions viewed as essential to success of social prescribing programmes

We identified three core elements seen as central in delivering social prescribing initiatives, along with internal and external influences that were important for their successful implementation and longevity (Fig. 2). These core elements and influences are discussed below.

- Core Element 1: A personalised approach

Operationalising a ‘personalised approach’ was central to social prescribing programmes represented in the workshop; a concept which in practice involved developing a detailed knowledge of and relationship with the community resources available, having person-centred conversations with service-users about what is important to them, and matching needs and interests to the resources available. This occurred in all programmes regardless of their discourse. The role of the link worker or similar in supporting this process was prominent within both the workshop and survey. This conforms to NHS England’s model of what ‘good social prescribing’ looks like and the vision for delivery of personalised care across services [15].

- Core Element 2: Public and service-user involvement

Public and service-user involvement and engagement was viewed as important for blending knowledge across traditional divides, supporting a move towards more holistic services and ensuring that services are adaptable to changing local needs. Workshop attendees discussed the need for more public involvement in the design and development of local community resources. Important benefits of involving service users as co-producers and designers of SP services have been highlighted previously. Thomas et al [32] note in particular service users/advisors’ importance for early identification of potential challenges to engagement with services and tailoring services to meet patients’ needs. They also highlight benefits to service users’ self-confidence and mood linked to their sense of control, being valued and listened to which encouraged their active involvement with services. A recent systematic review of social prescribing interventions targeting mental health found ‘no explicit evidence’ of service-user involvement in the co-design of social prescribing interventions, with authors noting potential implications for individual acceptability and engagement with such interventions [33]. However, 19/50 (38%) of the social prescribing programme surveys reported public involvement in co-creation of services, suggesting there may have been some recent progress in this direction. This is a particularly important finding given the noted patient wellbeing benefits of co-design and co-produced of SP intervention [32].

- Core element 3: Whole systems working

Workshop attendees discussed the importance of collaborative working across services to deliver holistic support to meet the social, emotional and psychological needs of local populations. Colocation of some services was identified as supporting this collaborative endeavour. Other authors [32] have highlighted how colocation can foster close relationships between different sectors, which authors view as essential for sustaining co-production and engendering trust in services and continuing referral. This is also a key element described within the NHS model of social prescribing ‘collaborative commissioning and partnership working’  [34].

### Internal influence 1: capacity

Workshop attendees identified various issues relating to capacity within the social prescribing service and system as a whole. This seems particularly pertinent given the expectation that a link worker may have an annual caseload of up to 250 people who they will work with for up to 3 months [35]. Furthermore, service-users with more complex needs are likely to need more extended and intensive support [35]. In the context of an upsurge in demand for social prescribing following the COVID-19 pandemic [36], there are fears that services could ‘shatter’ with recent evidence suggesting that up to a third of link workers are considering resigning [37]. Sandhu et al. [31] note the issue of limited numbers of link workers within local systems with some workers being shared across practices and Primary Care Networks. These authors also highlight serious limitations in support structures (management and training) for link workers. Although our workshop was held prior to the pandemic, the issue of capacity within local systems was already evident to the attendees. Key challenges identified included access to grants and other funding sources, recruiting and retaining volunteers, and lack of support for services from Local Authorities. This mirrors the findings of Thomas et al. [32] who identified capacity and resource issues as a key challenge to collaboration between GP staff and voluntary sector. The potential issue of recruitment and retention of volunteers and the need for financial investment and other types of support (training) to develop local resources was identified during workshop discussions. Such investment is important if we are to avoid disengagement with social prescribing through lack of availability of suitable and accessible local resources[35]. In a review of 22 social prescribing programmes, Sandhu et al. [31] found that none of the programmes measured impact on community groups. The expectation is that the providers of the social prescribing interventions are funded from charities and fundraising rather than the NHS budget and largely relies on communities and volunteering [15].

### Internal influence 1: evaluation

The wide variation in the evaluation of social prescribing initiatives and the difficulty of identifying appropriate outcome measures are already well documented [10, 22, 23, 31] and confirmed by our findings. Two-thirds of survey respondents who had evaluated a social prescribing programme highlighted the challenge of determining appropriate outcomes and measurement of outcomes. Whilst it has been recognised that diversity in social prescribing brings with it a diversity of approaches to evaluation, use of established validated measures (e.g. WEMWEBS) that allow comparison across services continues to be advocated [15]. Workshop attendees discussed the conundrum of increasing pressure to use validated outcome measures when bespoke but non-validated measures might be more appropriate to the objectives of the programme. It was noted that realisation of impact might require a more longitudinal approach that sits less well with result-driven commissioning and the requirement to demonstrate effectiveness within a shorter timeframe (Table 2). It has been argued previously that evidencing the full range of potential improvements due to social prescribing services are likely to require a broad range of data collection methods and evaluation over more extended periods of time [36]. Thomas et al. [32] noted the importance of evaluation in the sustainability of coproduction, however highlight likely challenges associated with this, including a tendency for GPs to require quantifiable outcomes as demonstration of effectiveness, whilst voluntary sector organisations often have a preference for qualitative evaluation.

### External influence 1: funding quantity and continuity

The need for more and longer-term financial investment in social prescribing was also identified, and the majority of survey respondents reported a reliance on short-term funding. This supports calls by GPs for more resources to support social prescribers [36]. Thomas et al. [32] note that the limited and short-term funding of many voluntary providers could create ‘unintended unreliability’, which could influence social prescribers’ willingness to refer to voluntary providers, with further implications for future funding. Sandhu et al.[31] highlight funding of SP services as one of a number of priority areas for both practitioners and researchers.

### External influence 2: stakeholder support

Workshop attendees discussed the need for stakeholder support, especially from commissioners, and coordination of local resources. Given the multifaceted nature of social prescribing local leadership was considered important in delivering a coordinated approach. The importance of effective leadership in advocating for mutuality between the co-producers of social prescribing services has been highlighted previously [32]. NHS England guidance also highlights the importance of investment in local resources through a range of mechanisms including collaborative commissioning and provision of start-up grants [15]. Outcomes-based commissioning has also been suggested as particularly suited to co-commissioning between health and social care [15]. Such an approach requires stakeholders to work together to agree appropriate outcomes. However, it has been noted previously that, where stakeholders hold very different theoretical perspectives, it is unlikely that outcomes data deemed appropriate by all can be collected [22].

### Strengths and limitations of this analysis

This analysis aimed to identify some of the key factors necessary for the implementation and delivery of a successful social prescribing programme, and some of the key challenges for implementation and delivery, from the perspective of people working on the implementation and delivery of social prescribing programmes in the North West Coast area of England. We drew on two sources of data; a ‘learning exchange’ workshop of people who had been involved in implementation projects or evaluations of social prescribing programmes supported by CLAHRC NWC, and an on-line survey open to anybody currently involved in social prescribing in NWC area. We used mixed methods to collate, synthesise and interpret the data. Based on our analysis, we present a visual representation of the essential core elements, influences, and resources needed (Fig. 2).

Probably the greatest strength of this analysis is the inclusion of mixed qualitative and quantitative methods. The workshop provided space and time for open discussion of which factors were seen as important and what the main challenges were. This information from the workshop informed the questions included within the later survey, which aimed to check for wider applicability and quantify some of these issues.

Most of the workshop attendees had previously been involved in CLAHRC-supported implementation or evaluation projects. This experience provided them with the time and space to consider what their social prescribing programme needed, thereby preparing them to share these insights at the workshop. However, it may also have provided them with more time and space, compared to many others working in the field, to read and consider the relevant social prescribing literature. This may have influenced their perceptions or communication of what constitutes ‘good social prescribing practice’ towards what they may have perceived the researchers and other workshop attendees to ‘want to hear’.

The inclusion of Public Advisors in both the original CLAHRC projects and the workshop ensured the views of the public (i.e. potential service-users) were incorporated. However, their presence in the groups, together with the CLAHRC NWC expectation of public involvement in all research, might also have influenced the identification of ‘public involvement’ as a key element of successful social prescribing programmes.

As the research team did not have access to a list of all social prescribing programmes across the North West Coast area, we sent survey links to our known contacts and asked them to forward the link to others working in social prescribing (snowballing). As we did not have a known sampling frame, the response rate is unknown, and it is not possible to estimate or describe response bias. This limits the generalisability of the findings.

The survey was also adversely affected by the COVID-19 pandemic. The survey link was distributed in February 2020, only a few weeks before the first COVID-19 ‘lockdown’, after which many social prescribers became extremely busy adapting services to remote working and supporting ‘shielded’ patients. This probably reduced the number of responses received. We had also hoped to consolidate our findings through a wider ‘Social Prescribing Knowledge Exchange’ event, where we would present the findings of the workshop and survey and facilitate further discussions based on those findings. The event was planned for April 2020 and had around 180 people registered to attend, but had to be cancelled and proved impossible to rearrange through the pandemic.

As most of the data were collected before the COVID-19 pandemic had significantly affected the UK, this analysis does not capture the its influence, which may have had lasting impacts on social prescribing in the region. In addition, there are now social prescribing link workers in all Primary Care Networks in England, and social prescribing is being actively encouraged and commissioned [34]. It should also be noted that all of the data was collected within the North West Coast area of England, and the findings may have been slightly different in other areas of the UK. There may also be important differences in context between the UK and other countries where social prescribing is practised.

### What this analysis adds to the existing knowledge base

This analysis tends to confirm the findings of other published studies and recommendations of best practice guidelines, from the point of view of people working to implement and delivery social prescribing programmes in the NWC area of England. To our knowledge, we are the first authors to attempt to summarise the key considerations for a successful social prescribing programme as a visual representation.

### Implications for practice

This analysis confirms the need to involve patients and members of the public in co-design and coproduction of the service, to adopt a person-centred approach, for social prescribing to be integrated within whole systems, and to adequately evaluate what has been achieved. It also confirms the need for stakeholder support and provision of adequate, stable resources for both social prescribing (the action of supporting people to access local community resources and activities to improve their wellbeing) and the community resources they rely on.

The problems identified in securing adequate funding and resources may have intensified since this research was conducted. A recent report, based on information and research undertaken during the early months of the pandemic, identified a greater need for services provided by community groups than before lockdown. This need was set within a context of loss of income for four in five groups and fewer volunteers during lockdown. Groups were less confident about being able to continue in the future than they were before the pandemic, with access to funding in both short and long term identified as most important [38, 39].

With the widespread introduction of primary care-based link workers, there will be a need to co-ordinate and collaborate with existing social prescribing services to harness knowledge and avoid duplication of effort.

### Implications for research and evaluation

It has been noted previously that whilst there has been a shift towards a biopsychosocial model of health, what constitutes valuable evidence of success in care delivery continues to be underpinned by a medical model of health rooted in scientific quantification [22]. It has also been suggested that the shift to a biopsychosocial model of health offers an opportunity to re-think how the impact of social prescribing is measured [22]. Based on our findings such a re-think would be welcomed including a greater focus on measuring impact on the wider determinants of health [22].

There is increasing support for taking a more nuanced and contextualised approach to measure effectiveness of social prescribing and recognition of the need for use of more realistic and relevant outcomes [10, 23]. Key questions remain on how to measure success and effectiveness effectively and consistently across social prescribing initiatives, with outcome measures covering not just individual, but service (including impact on other health and social services and organisations), community and societal outcomes [31, 40]. Sandhu et al. [31] identify priority areas of focus, including mapping of onward referrals; comparisons of different models of programme structure and funding; evaluations of comparative effectiveness among patient groups; considerations of strategies for maximising referrals; and explorations of staff training and supervision.

The suggestion from workshop attendees to improve data sharing across projects to obtain better evidence of effectiveness will be a topic of further exploration by ARC NWC.

## Conclusion

The model presented in this paper provides a visual representation of the key elements for successful implementation and sustainability of social prescribing programmes, as highlighted by practitioners working within social prescribing and interpreted by researchers at a point in time when social prescribing was starting to be disseminated throughout the NHS. Further application of this model across different contexts will allow the model to be refined and become more generalisable.

## Supplementary Information

**Additional file 1.**

## Data Availability

All data generated or analysed during this study are included in this published article and its supplementary information files.
