# Supplementary material for: Social prescribing practices and learning across the North West Coast region: essential elements and key challenges to implementing effective and sustainable social prescribing services
Source: BMC Health Serv Res. 2023 May 31;23:562. doi: 10.1186/s12913-023-09574-6 (PMC10231851; doi:10.1186/s12913-023-09574-6)
Supplement: Supplementary file 1 — Additional file 1. [file 12913_2023_9574_MOESM1_ESM.pdf]

NIHR | Applied Research Collaboration North West Coast

Social Prescribing Survey

Introduction

Please take a few minutes to complete this survey. This survey is designed to capture a snapshot of social prescribing activities across the North West Coast region and also to highlight the challenges. It should only take you 10-15 mins to complete! Questions marked with a * are required. The survey link is re-usable - please forward the link to anybody else who you know is involved in social prescribing within the region.

*Required

Why are we doing this survey?

We firstly want to capture information on the range of social prescribing activities currently being undertaken within the North West Coast area. The findings will be used to inform the research strategy for social prescribing for National Institute for Health Research Applied Research Collaboration North West Coast (NIHR ARC NWC). Secondly, we value your experience and your suggestions of research questions also with a view of scoping who may want to work collaboratively on social prescribing within. Thirdly, this survey will reflect a baseline of what Social Prescribing activities are being done by NIHR ARC NWC members and within the region. Finally, we will do two follow-up surveys, midway and at the end of the NIHR ARC NWC, to show progression and impact of Social Prescribing activities over time. For more information about the NIHR ARC NWC please follow this link: https://arc-nwc.nihr.ac.uk.

What is social prescribing?

Social prescribing aims to help people change social factors (e.g. financial problems, social isolation) that are negatively affecting their health. It usually involves working with a person to identify what is important to them, and then linking them into appropriate services and activities in their local community. Examples might include housing support, welfare benefits advice, volunteering opportunities, or social activities. Projects using a social prescribing approach are not always labelled as 'social prescribing' – for example they may be referred to instead as 'community referral', 'a holistic approach' or 'a social approach'. For the purposes of this survey, 'social prescribing' may refer to the process of linking people in with local services and activities, providing these as part of a social prescribing programme, or both.

Why have I received a survey link?

We have sent this survey link to ARC NWC partners and other people within the North West Coast area who we know are involved in social prescribing. We have also asked people who receive a survey link to forward the link onto others who are involved in social prescribing in the region.

How we will use your data

In addition to your information collected in this survey, we will also collect your email address (optional) to be able to contact you for follow up if needed. We securely store this survey data until the end of 2024, when the NIHR ARC NWC programme will be over. An anonymised summary of the findings will be published on the ARC NWC website (https://arc-nwc.nihr.ac.uk/) and emailed to all respondents who provided an email address. The anonymised findings will be presented at an ARC social prescribing knowledge event (29th April, Gujarat Centre, South Meadow Lane, Preston PR1 8JN).The anonymised findings may also contribute to a published paper and will be used for comparison with future survey findings.

A. **Do you wish to continue with the survey?** *

   Yes/No

Your experience of social prescribing

B. What organisation do you belong to?

C. If other, please let us know the organisation you belong to?

D. Are you ACTIVELY INVOLVED in social prescribing within your organisation or locality? *

Yes/No

(E.g. Link worker, Manager of linking service, Provider of activities, Commissioner of Social Prescribing services)

E. Please list the social prescribing projects you are ACTIVELY INVOLVED in.

List by title

Please provide more details about the above social prescribing projects that you are ACTIVELY INVOLVED with.

You can describe up to 3 projects within this survey. If you are involved in more than 3 projects, please start another survey, or forward the survey link onto somebody else who could provide that information.

First Project

Please provide details of the First Project you are involved with.

1.1: Title of this Project

1.2: How are you involved with this project? (Please tick all that apply)

a) Link worker or similar role (e.g. Community Navigator or Connector)
b) Manager of Linking Services
c) Provider of Activities
d) Commissioner of Social Prescribing Services
e) Other (please specify)

1.3: Project Aims

1.4: Type of Project (Please tick all that apply)

a) Linking people to activities
b) Running activities
c) Other (please specify)

1.5: Who does the linking roles in your project? (Please tick all that apply)

a) Link Worker
b) Community Navigator
c) Community Connector
d) Not Applicable
e) Other (please specify)

1.6: How do service users access this project? (Please tick all that apply)

a) Self-referral
b) Drop-in
c) Primary care - GP Practices
d) Community Health Care
e) Social Care
f) Secondary Care - Physical Health
g) Secondary Care - Mental Health

h) Other (please specify)

1.7: What type of area(s) is/are covered by this project? (Please tick all that apply)

a) Urban
b) Rural
c) Semi-urban
d) Other (please specify)

1.8: Where is this projected located?

1.9: Population groups served (general) (Please tick all that apply)

a) Children
b) Young people
c) Working age
d) Retirement age/Elderly
e) Other (please specify)

1.10: Does this project focus on particular groups? (Please tick all that apply)

a) Male
b) Female
c) Pregnant women or New mothers
d) Families with young children
e) Carers
f) People with a specific condition or disability
g) People with specific cultural needs (e.g. Ethnic Minorities, LGBT)
h) Other (please specify)

1.11_i: Is the intervention time-bound/time-limited?

Yes/No

1.11_ii: If yes, please provide details

1.12_i: Does the project use motivational techniques to support and encourage service users to attend activities?

For example, calling them up, accompanying them to activities, psychological methods, etc.

Yes/No

1.12_ii: If yes, please provide details

1.13_i: Is there public involvement in this project?

Yes/No/Don't Know

1.13_ii: If yes, how are they involved? (Please tick all that apply)

a) Co-creation
b) Delivery
c) Modifications
d) Evaluating
e) Monitoring
f) Raising awareness
g) Other (please specify)

1.14: How long has this project been running? (Mark one only)

a) 0-6 months

b) 6-12 months
c) 1-3 years
d) >3 years
e) Don't Know

1.15: How is this project funded? (Please tick all that apply)

a) Commissioned Service
b) Internal funding
c) External Grant funding
d) Other (please specify)

1.16: How long is this project funded for? (Mark one only)

From the start of the project.

a) Less than 2 years
b) 2 to 5 years
c) More than 5 Years
d) No specific time limit
e) Don't Know
f) Other (please specify)

1.17: What have been the key challenges to the success of the project? (Please tick all that apply)

a) Securing adequate and sustainable funding
b) Getting the programme set up and running
c) Developing skills or capacity – linking people into activities
d) Developing skills or capacity – community support or activities
e) Engaging or motivating people to engage in services or activities
f) Not applicable – no major challenges
g) Other (please specify)

1.18: Would you like to add any additional comments about the challenges you identified?

1.19: Are there any specific challenges or questions relating to the running of your project that you would like to find the answers to?

1:20_i: Are you doing an evaluation of this project?

Yes/No/Other (please specify)

1.20_ii: What methods are you using in your evaluation?

1.20_iii: What have been the key challenges of evaluating your project?

a) Deciding what outcomes to measure or data to collect
b) Capturing or accessing the data you need
c) Analysing the data
d) Interpreting the fi ndings
e) Making changes based on the fi ndings
f) Finding the time for evaluation
g) Satisfying the funders' reporting requirements
h) Not applicable – no major challenges
i) Other (please specify)

1.21: Are there any specific challenges or questions relating to the evaluation of your project that you would like to find the answers to?

1.22: Is there anything else you would like to tell us about this project, that is not covered by the answers above?

F. **Are you also involved in another social prescribing project?** *

Yes/No

If YES, you will be asked to provide details of this second project.

Second Project

Please provide details of the Second Project you are involved with.

2.1: Title of this Project

2.2: How are you involved with this project? (Please tick all that apply)

f) Link worker or similar role (e.g. Community Navigator or Connector)
g) Manager of Linking Services
h) Provider of Activities
i) Commissioner of Social Prescribing Services
j) Other (please specify)

2.3: Project Aims

2.4: Type of Project (Please tick all that apply)

d) Linking people to activities
e) Running activities
f) Other (please specify)

2.5: Who does the linking roles in your project? (Please tick all that apply)

f) Link Worker
g) Community Navigator
h) Community Connector
i) Not Applicable
j) Other (please specify)

2.6: How do service users access this project? (Please tick all that apply)

i) Self-referral
j) Drop-in
k) Primary care - GP Practices
l) Community Health Care
m) Social Care
n) Secondary Care - Physical Health
o) Secondary Care - Mental Health
p) Other (please specify)

2.7: What type of area(s) is/are covered by this project? (Please tick all that apply)

e) Urban
f) Rural
g) Semi-urban
h) Other (please specify)

2.8: Where is this projected located?

2.9: Population groups served (general) (Please tick all that apply)

f) Children

g) Young people
h) Working age
i) Retirement age/Elderly
j) Other (please specify)

2.10: Does this project focus on particular groups? (Please tick all that apply)

i) Male
j) Female
k) Pregnant women or New mothers
l) Families with young children
m) Carers
n) People with a specific condition or disability
o) People with specific cultural needs (e.g. Ethnic Minorities, LGBT)
p) Other (please specify)

2.11_i: Is the intervention time-bound/time-limited?

Yes/No

2.11_ii: If yes, please provide details

2.12_i: Does the project use motivational techniques to support and encourage service users to attend activities?

For example, calling them up, accompanying them to activities, psychological methods, etc.

Yes/No

2.12_ii: If yes, please provide details

2.13_i: Is there public involvement in this project?

Yes/No/Don’t Know

2.13_ii: If yes, how are they involved? (Please tick all that apply)

h) Co-creation
i) Delivery
j) Modifications
k) Evaluating
l) Monitoring
m) Raising awareness
n) Other (please specify)

2.14: How long has this project been running? (Mark one only)

f) 0-6 months
g) 6-12 months
h) 1-3 years
i) >3 years
j) Don't Know

2.15: How is this project funded? (Please tick all that apply)

e) Commissioned Service
f) Internal funding
g) External Grant funding
h) Other (please specify)

2.16: How long is this project funded for? (Mark one only)

From the start of the project.

g) Less than 2 years
h) 2 to 5 years
i) More than 5 Years
j) No specific time limit
k) Don't Know
l) Other (please specify)

2.17: What have been the key challenges to the success of the project? (Please tick all that apply)

h) Securing adequate and sustainable funding
i) Getting the programme set up and running
j) Developing skills or capacity – linking people into activities
k) Developing skills or capacity – community support or activities
l) Engaging or motivating people to engage in services or activities
m) Not applicable – no major challenges
n) Other (please specify)

2.18: Would you like to add any additional comments about the challenges you identified?

2.19: Are there any specific challenges or questions relating to the running of your project that you would like to find the answers to?

2.20_i: Are you doing an evaluation of this project?

Yes/No/Other (please specify)

2.20_ii: What methods are you using in your evaluation?

2.20_iii: What have been the key challenges of evaluating your project?

j) Deciding what outcomes to measure or data to collect
k) Capturing or accessing the data you need
l) Analysing the data
m) Interpreting the fi ndings
n) Making changes based on the fi ndings
o) Finding the time for evaluation
p) Satisfying the funders' reporting requirements
q) Not applicable – no major challenges
r) Other (please specify)

2.21: Are there any specific challenges or questions relating to the evaluation of your project that you would like to find the answers to?

2.22: Is there anything else you would like to tell us about this project, that is not covered by the answers above?

G. Are you also involved in another social prescribing project? *

Yes/No

If YES, you will be asked to provide details of this third project.

Third Project

Please provide details of the Third Project you are involved with.

3.1: Title of this Project

3.2: How are you involved with this project? (Please tick all that apply)

k) Link worker or similar role (e.g. Community Navigator or Connector)
l) Manager of Linking Services
m) Provider of Activities
n) Commissioner of Social Prescribing Services
o) Other (please specify)

3.3: Project Aims

3.4: Type of Project (Please tick all that apply)

g) Linking people to activities
h) Running activities
i) Other (please specify)

3.5: Who does the linking roles in your project? (Please tick all that apply)

k) Link Worker
l) Community Navigator
m) Community Connector
n) Not Applicable
o) Other (please specify)

3.6: How do service users access this project? (Please tick all that apply)

q) Self-referral
r) Drop-in
s) Primary care - GP Practices
t) Community Health Care
u) Social Care
v) Secondary Care - Physical Health
w) Secondary Care - Mental Health
x) Other (please specify)

3.7: What type of area(s) is/are covered by this project? (Please tick all that apply)

i) Urban
j) Rural
k) Semi-urban
l) Other (please specify)

3.8: Where is this projected located?

3.9: Population groups served (general) (Please tick all that apply)

k) Children
l) Young people

m) Working age
n) Retirement age/Elderly
o) Other (please specify)

3.10: Does this project focus on particular groups? (Please tick all that apply)

q) Male
r) Female
s) Pregnant women or New mothers
t) Families with young children
u) Carers
v) People with a specific condition or disability
w) People with specific cultural needs (e.g. Ethnic Minorities, LGBT)
x) Other (please specify)

3.11_i: Is the intervention time-bound/time-limited?

Yes/No

3.11_ii: If yes, please provide details

3.12_i: Does the project use motivational techniques to support and encourage service users to attend activities?

For example, calling them up, accompanying them to activities, psychological methods, etc.

Yes/No

3.12_ii: If yes, please provide details

3.13_i: Is there public involvement in this project?

Yes/No/Don’t Know

3.13_ii: If yes, how are they involved? (Please tick all that apply)

o) Co-creation
p) Delivery
q) Modifications
r) Evaluating
s) Monitoring
t) Raising awareness
u) Other (please specify)

3.14: How long has this project been running? (Mark one only)

k) 0-6 months
l) 6-12 months
m) 1-3 years
n) >3 years
o) Don't Know

3.15: How is this project funded? (Please tick all that apply)

i) Commissioned Service
j) Internal funding
k) External Grant funding
l) Other (please specify)

3.16: How long is this project funded for? (Mark one only)

From the start of the project.

m) Less than 2 years
n) 2 to 5 years
o) More than 5 Years
p) No specific time limit
q) Don't Know
r) Other (please specify)

3.17: What have been the key challenges to the success of the project? (Please tick all that apply)

o) Securing adequate and sustainable funding
p) Getting the programme set up and running
q) Developing skills or capacity – linking people into activities
r) Developing skills or capacity – community support or activities
s) Engaging or motivating people to engage in services or activities
t) Not applicable – no major challenges
u) Other (please specify)

3.18: Would you like to add any additional comments about the challenges you identified?

3.19: Are there any specific challenges or questions relating to the running of your project that you would like to find the answers to?

3.20_i: Are you doing an evaluation of this project?

Yes/No/Other (please specify)

3.20_ii: What methods are you using in your evaluation?

3.20_iii: What have been the key challenges of evaluating your project?

s) Deciding what outcomes to measure or data to collect
t) Capturing or accessing the data you need
u) Analysing the data
v) Interpreting the fi ndings
w) Making changes based on the fi ndings
x) Finding the time for evaluation
y) Satisfying the funders' reporting requirements
z) Not applicable – no major challenges
aa) Other (please specify)

3.21: Are there any specific challenges or questions relating to the evaluation of your project that you would like to find the answers to?

3.22: Is there anything else you would like to tell us about this project, that is not covered by the answers above? *

If you are involved in more than these 3 projects, please start another social prescribing survey, or forward the survey link onto somebody else who could provide that information.

H. Are you AWARE OF any social prescribing projects within your organisation or locality?

Yes/No

I. Please list the social prescribing projects you are AWARE OF but not actively involved with.

List by title.

NOTE: If you have contact details for anyone ACTIVELY INVOLVED in the projects above, please forward them this Social Prescribing survey link.

Research experience

J. **Are you already involved in social prescribing research?**

Yes/No

K. **If 'yes', what is/are the title(s) of your ongoing project(s)?**

L. **How are you involved with this research? (Please tick all that apply)**

a) Link worker or similar role
b) Manager of Linking Services
c) Provider of Activities
d) Commissioner of Social Prescribing
e) Researcher
f) Public and Patient Adviser
g) Other (please specify)

Future Activities

M. **Please provide us with a contact email below if you tick YES to any of these questions.**

a) Would you be interested in participating in a potential joint research collaboration? Yes/No
b) We are organising acknowledge exchange event on the 29thApril 2020 – would you be interested in attending? Yes/No
c) Would you like us to keep you updated on other social prescribing events reorganise? Yes/No

N. **Email Address**

This is optional. An anonymised summary of the findings of this Social Prescribing survey will be sent to all respondents who provide an email address. We take your privacy very seriously, and your email will be kept confidential. We will only use your email used only to contact you about NIHR ARC NWC social prescribing-related activities in the area.
